# The impact of post intensive care syndrome in patients surviving the ICU: the downside of ICU treatment

**DOI:** 10.1186/2197-425X-3-S1-A530

**Published:** 2015-10-01

**Authors:** D Ramnarain, A Rutten, G Van der Nat, J Van Gorp, I Gnirrep, S Voermans-Schellekens, W Schapendonk, C Van Slobbe, L Savelsberg-Huijbregts, I Schoenmakers-Verheijden, N Van der Lely

**Affiliations:** Elisabeth Tweesteden Hospital Tilburg, Intensive Care Medicine, Tilburg, the Netherlands; Elisabeth Tweesteden Hospital Tilburg, Tilburg, the Netherlands

## Introduction

Despite reduced mortality and increasing survival rate of ICU treatment, a large group of patients surviving the ICU have a variety of complaints. Survivors of critical illness can undergo dramatic changes in their lives as a result of their experience, with many having some form of deficit in one or more domains of physical, psychological or cognitive functioning. There is still much to learn about the magnitude of the so-called Post ICU Syndrome (PICS) in patients surviving in the ICU.

## Objectives

We studied the incidence of somatic complaints, anxiety, depression and Post-traumatic stress disorder (PTSD) in ICU survivors.

## Methods

We screened for somatic complaints, anxiety, depression and PTSD in patients who were treated in our ICU from January first 2013 until December 31 2013 for more than 5 days. These patients were invited to visit our post-ICU aftercare clinic. Six weeks after discharge a letter of invitation together with health related questionnaire, hospital anxiety and depression scale (HADS) questionnaire, and IES-R questionnaire for screening for PTSD, was send. All data were analysed and if treatment was indicated patients were referred for further treatment.

## Results

A total of 316 patients were admitted to the ICU for more than 5 days. Fifty four patients were excluded because of various reasons, 25 patients died after ICU discharge. A total of 97 patients visited our post ICU after care. Seventy patients (81.4%) had somatic complaints influencing daily performance and quality of life. Most frequently reported complaints were; fatigue (74.4%), muscle weakness (48.8%), dyspnoea (34.9%), impairment of daily activity (81.4%), pain (38.4%) and weight loss (33.3%). Pain was most reported in patients with SAH (27.3%), multi-trauma (15.2%) and pneumonia (12.1%). There was no significant difference in somatic complaints between men and women.

Clinically significant anxiety and/or depression was seen in 45.4%. Women compared to men showed significant more HAD symptoms (26.8 vs 18.6 %, p < 0.05). Patients with SAH, neurotrauma and multi-trauma patients showed more clinically significant HAD symptoms. No significant association between age, diagnosis at ICU admission, length of stay ICU, severity of illness, delirium and use of sedatives, and HAD was seen.

Prevalence of PTSD was 43.3% and most seen in patients with SAH. Women compared to men reported significantly more PTSD. Pain, muscle weakness, fatigue, impairment in daily activity, dyspnoea and hoarseness was significantly associated with HADS and PTSD.

## Conclusions

Many patients surviving the ICU have somatic complaints, anxiety and/or depression or PTSD influencing daily performance. It is of great importance to recognize and treat various complaints in an early stage after ICU survival in order to improve quality of life in ICU survivors.

